# Supplementary material for: Microrobot with Gyroid Surface and Gold Nanostar for High Drug Loading and Near-Infrared-Triggered Chemo-Photothermal Therapy
Source: Pharmaceutics. 2022 Nov 6;14(11):2393. doi: 10.3390/pharmaceutics14112393 (PMC9696578; doi:10.3390/pharmaceutics14112393)
Supplement: Supplementary file 1 [file pharmaceutics-14-02393-s001.zip › pharmaceutics-1997329-supplementary.pdf]

pharmaceutics

MDPI

Supplementary material

Microrobot with Gyroid Surface and Gold Nanostar for High Drug Loading and Near-Infrared-Triggered Chemo-Photothermal Therapy

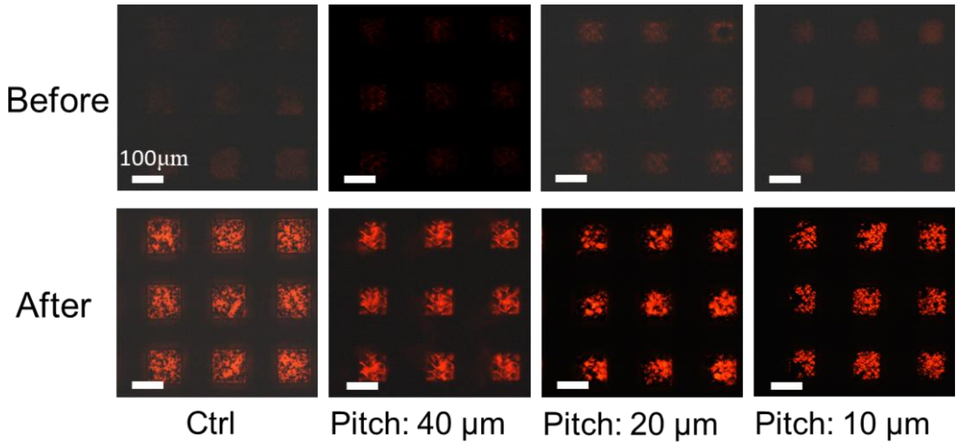

Figure S1. Fluorescence images of solid cubic and different types of GS-MCs before and after drug loading.

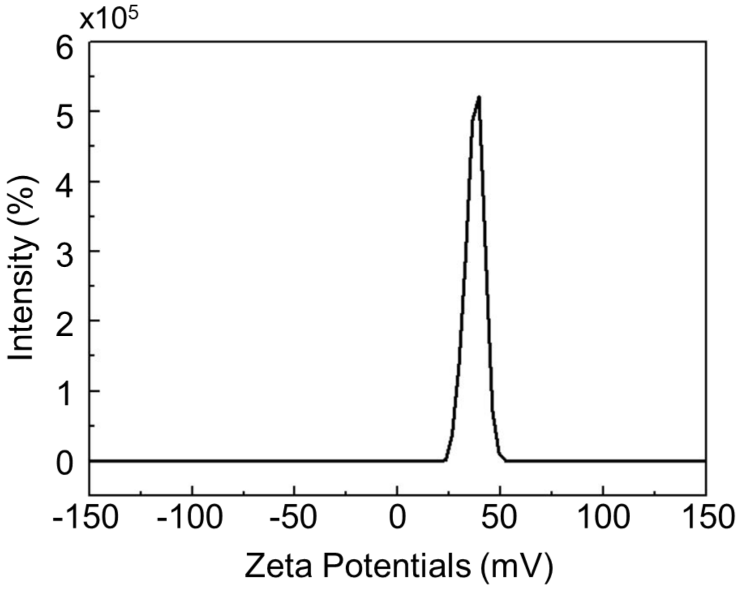

Figure S2. Zeta potential graph showing that the ionic surface charge of MNPs is positive.

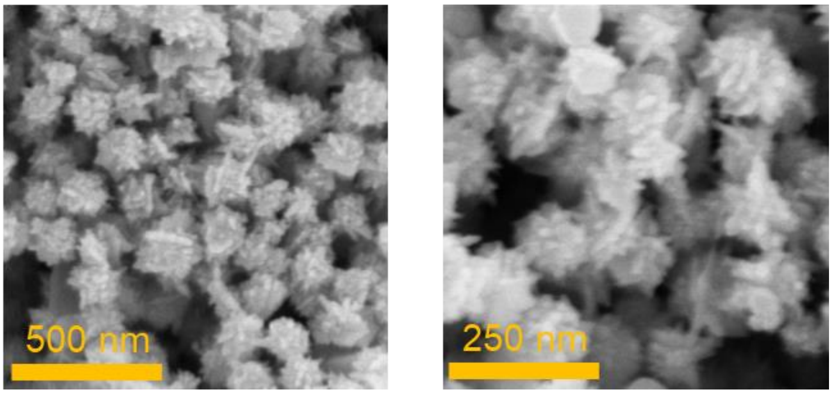

Figure S3. SEM images of the Au-nanostar.

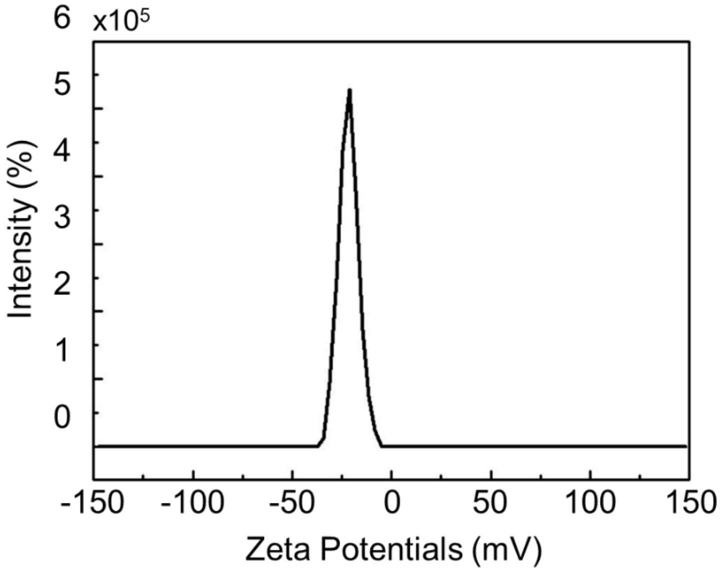

Figure S4. Zeta potential graph showing that the ionic surface charge of Au-nanostar is negative.

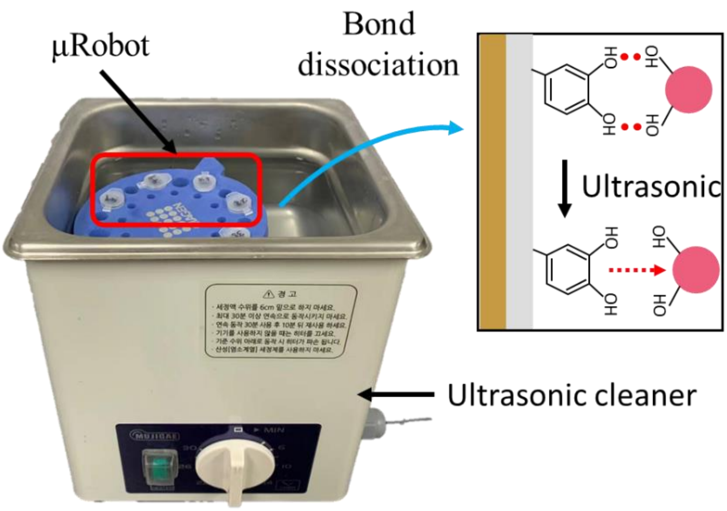

Figure S5. GS-HMs placed in an EP tube and in a ultrasonic cleaner to dissociate the noncovalently bonded doxorubicin (DOX) molecules.

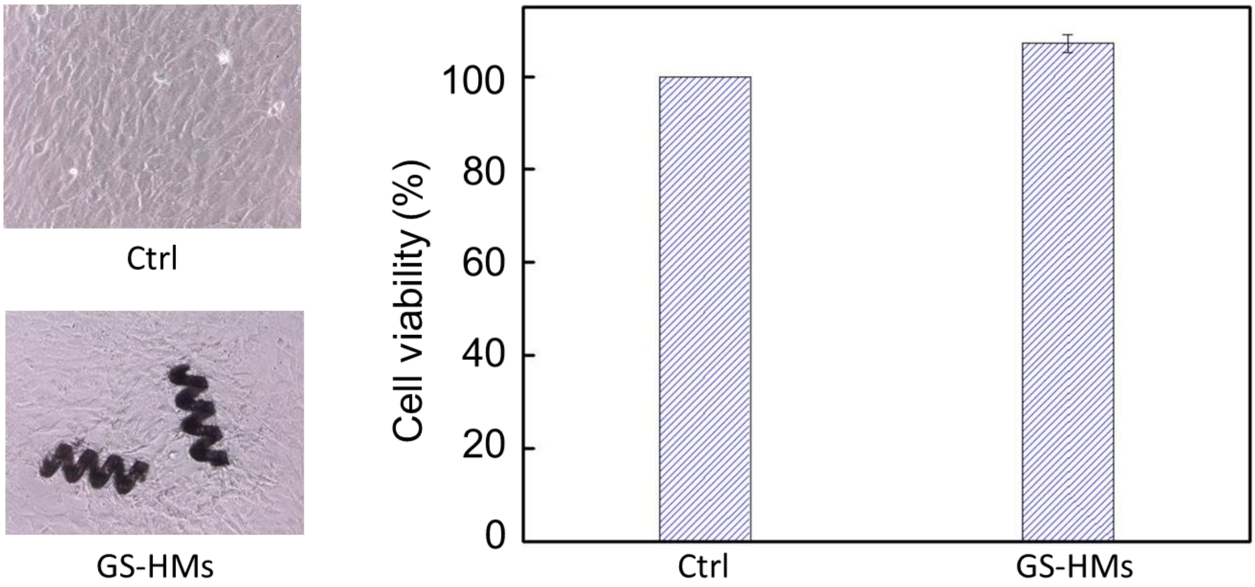

Figure S6. Cell viability of GS-HMs against normal cells.

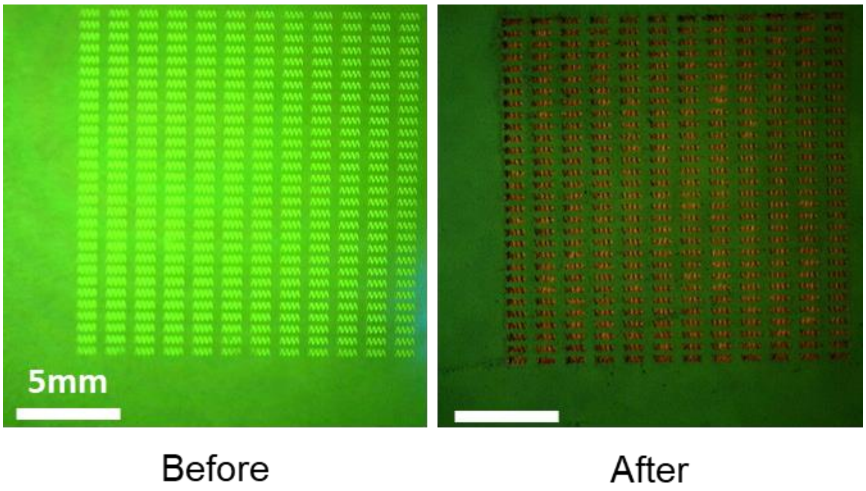

Figure S7. Pen microscopy fluorescence images before and after DOX loading into the GS-HMs.
